# Glycinergic transmission modulates GABAergic inhibition in the avian auditory pathway

**DOI:** 10.3389/fncir.2014.00019

**Published:** 2014-03-14

**Authors:** Matthew J. Fischl, R. Michael Burger

**Affiliations:** Department of Biological Sciences, Lehigh UniversityBethlehem, PA, USA

**Keywords:** glycine, GABA, inhibition, cross-suppression, interaural time disparities

## Abstract

For all neurons, a proper balance of synaptic excitation and inhibition is crucial to effect computational precision. Achievement of this balance is remarkable when one considers factors that modulate synaptic strength operate on multiple overlapping time scales and affect both pre- and postsynaptic elements. Recent studies have shown that inhibitory transmitters, glycine and GABA, are co-released in auditory nuclei involved in the computation of interaural time disparities (ITDs), a cue used to process sound source location. The co-release expressed at these synapses is heavily activity dependent, and generally occurs when input rates are high. This circuitry, in both birds and mammals, relies on inhibitory input to maintain the temporal precision necessary for ITD encoding. Studies of co-release in other brain regions suggest that GABA and glycine receptors (GlyRs) interact via cross-suppressive modulation of receptor conductance. We performed *in vitro* whole-cell recordings in several nuclei of the chicken brainstem auditory circuit to assess whether this cross-suppressive phenomenon was evident in the avian brainstem. We evaluated the effect of pressure-puff applied glycine on synaptically evoked inhibitory currents in nucleus magnocellularis (NM) and the superior olivary nucleus (SON). Glycine pre-application reduced the amplitude of inhibitory postsynaptic currents (IPSCs) evoked during a 100 Hz train stimulus in both nuclei. This apparent glycinergic modulation was blocked in the presence of strychnine. Further experiments showed that this modulation did not depend on postsynaptic biochemical interactions such as phosphatase activity, or direct interactions between GABA and GlyR proteins. Rather, voltage clamp experiments in which we manipulated Cl^−^ flux during agonist application suggest that activation of one receptor will modulate the conductance of the other via local changes in Cl^−^ ion concentration within microdomains of the postsynaptic membrane.

## Introduction

Inhibitory input plays an integral role in the maintenance of temporal precision in the avian sound localization circuit (Funabiki et al., 1998; Yang et al., 1999; Lu and Trussell, 2000; Monsivais et al., 2000; Fukui et al., 2010; Burger et al., 2011; Coleman et al., 2011). Recent work revealed a novel form of inhibition in this circuit that results from the co-release of GABA and glycine from the same vesicles. This mode of transmission occurs in some synapses at the nucleus angularis (NA; Kuo et al., 2009) and superior olivary nucleus (SON; Coleman et al., 2011) where GABA and glycine each account for approximately 50% of the total amplitude of evoked inhibitory postsynaptic currents (IPSCs). Glycinergic transmission was also observed in the nucleus magnocellularis (NM) and nucleus laminaris (NL), where stimulation at high but physiologically relevant rates evoked a slowly emerging glycinergic component of the inhibition (Fischl et al., 2014). This glycinergic component was functionally important, as blocking glycinergic transmission reduced the efficacy of inhibition in the NM. We have also shown that GlyR block reduced the ability of SON neurons to phase-lock to pure tone stimuli near best frequency *in vivo* (Coleman et al., 2011). Despite this recent progress, the function of glycine and its co-release with GABA is not well understood in this circuit.

Synaptic inhibition is a ubiquitous feature of neurons that process sound localization cues from the brainstem to the cortex. In both mammals and avians, these inputs are subject to modulatory mechanisms that confer plasticity to the strength of inhibition. These mechanisms influence both GABAergic and glycinergic synapses at either pre- or postsynaptic loci. Some of these mechanisms include suppression of release via GABA_B_R activation (Lu et al., 2005; Magnusson et al., 2008; Tang et al., 2009; Hassfurth et al., 2010; Takesian et al., 2010; Fischl et al., 2012) or metabotropic glutamate receptor activation (Lu, 2007; Tang et al., 2009), retrograde GABAergic signaling (Magnusson et al., 2008) and cannabinoid receptor activation (Trattner et al., 2013). Activation of various postsynaptic signaling cascades may also affect conductances in the postsynaptic cell (Kotak and Sanes, 2002, 2003; Chang et al., 2003). This striking diversity of mechanisms amongst various neurons along the auditory pathway suggests that modulation of inhibition is integral for processing at all levels of the system. In the avian brainstem, the recent discovery of functionally relevant glycinergic transmission warrants exploration of mechanisms that may shape this conductance and characterization of glycine and GABA interactions given their similar ion permeability.

Co-release of GABA and glycine originating from single vesicles is possible because these transmitters share a vesicular transport molecule (vesicular inhibitory amino acid transporter, VIAAT or VGAT; Burger et al., 1991; McIntire et al., 1997; Sagné et al., 1997; Wojcik et al., 2006). Loading of neurotransmitters into vesicles depends on their concentration in the axon terminals (Eulenburg et al., 2005; Apostolides and Trussell, 2013). Co-release of GABA and glycine in the mammalian auditory brainstem has been observed in developing neurons (Awatramani et al., 2005; Gillespie et al., 2005), however, in the avian brainstem, hallmarks of both GABA and glycinergic signaling persist at ages where synapses are considered to be mature (Fischl et al., 2014).

In other systems where both modes of transmission are present and proximal to one another, reception of GABA or glycine has been shown to modulate the complementary neurotransmitter’s action. Several experiments indicate that there is a cross-suppressive effect when both receptors are activated simultaneously. Studies in spinal cord neurons of rat (Li et al., 2003) and frog (Kalinina et al., 2009) indicate an asymmetry of occlusion where activation of GlyRs prior to GABAergic transmission yields a greater degree of suppression than the opposite condition (GABA preceding glycine). In one of these studies, the mechanism of this suppression was dependent on a signaling cascade involving phosphatase activity (Li et al., 2003). A study in rat olfactory bulb neurons showed a variety of occlusion phenotypes including neurons for which cross-suppression was either bi-directional, unidirectional, or absent (Trombley et al., 1999). Others have suggested that these results are a consequence of alteration in driving force by changes in Cl^−^ flux during receptor activation (Grassi, 1992; Karlsson et al., 2011). The wide range of observations regarding the cross-suppression between GABA and glycine suggests that the mechanisms involved may be specific to particular brain regions.

Given recent data suggesting that glycinergic transmission is more ubiquitous in the avian auditory circuitry than previously thought, we investigated how inhibitory synaptic transmission is affected by GlyR activation. We demonstrate that activation of GlyRs occludes synaptically evoked IPSCs in both NM and the SON. In our system, this interaction did not depend on phosphatase activity, but rather appeared to depend on local changes in Cl^−^ driving force. By manipulating or limiting the movement of Cl^−^ ions with voltage clamp, we show that ligand binding and activation of GlyRs is not sufficient to induce suppression. Further, by driving Cl^−^ into the neuron during glycine application (thereby increasing the Cl^−^ driving force) results in an enhanced evoked response. These data indicate that activation of GlyRs during inhibitory transmission provides an additional mechanism for modulation of inhibition and that titration of specific neurotransmitters at co-release terminals may influence synaptic integration at the postsynaptic membrane.

## Methods

All protocols and procedures were approved by the Lehigh University Institutional Animal Care and Use Committee.

### *In vitro* brain slice preparation

For *in vitro* physiology, 56 white leghorn chickens aged E17-P5 of either sex were rapidly decapitated and the brainstem containing auditory nuclei was removed, blocked, and submerged in oxygenated artificial cerebrospinal fluid (ACSF) (containing in mM: 130 NaCl, 3 KCl, 10 glucose, 1.25 NaH_2_PO_4_, 26 NaHCO_3_, 3 CaCl_2_, 1 MgCl_2_) at 22°C. The brainstem was placed rostral surface down on the stage of a vibrating microtome (HM650V, Microm). Coronal sections (150–200 µm) containing the auditory brainstem nuclei were collected, submerged in an incubation chamber of continuously oxygenated ACSF and incubated at 37°C for approximately 1 h. Slices were then maintained at room temperature until used for recording.

Brainstem slices were placed in a custom recording chamber on a retractable chamber shuttle system (Siskiyou Design Instruments) and neurons were visualized with a Nikon FN-1 Physiostation microscope using infrared differential interference contrast optics. Video images were captured using a CCD camera (Hammamatsu C7500-50) coupled to a video monitor. The recording chamber (volume ~1 ml) was continuously perfused with ACSF at a rate of 2–4 ml/min. An inline feedback temperature controller and heated stage were used to maintain chamber temperature at 35 ± 1°C (TC344B, Warner Instruments, Hamden, CT).

### *In vitro* whole-cell recordings

Patch pipettes were pulled from thick walled borosilicate glass capillary tubes (WPI 1B120F-4) to a resistance of 4–8 MΩ using a two-stage puller (Narishige PC-10, Tokyo, Japan) and back-filled with internal solution (containing in mM: 105 CsMeSO_3_, 35 CsCl, 5 EGTA, 10 HEPES, 1 MgCl_2_, 4 ATP-Mg, and 0.3 GTP-Na, pH 7.2 adjusted with KOH). 5 mM QX314 was added to the internal solution to prevent antidromic action potentials. In experiments where phosphatase 2B activity was blocked, cyclosporin A (0.5–1.5 µm) was added to the internal solution. In voltage clamp, series resistance was compensated at 60–80%. Membrane voltage was clamped using a Multiclamp 700B amplifier. The signal was digitized with a Digidata 1440 data acquisition board and recorded using Clampex software (Molecular Devices, Sunnyvale, CA).

### Effect of glycine receptor (GlyR) activation on inhibitory postsynaptic currents (IPSCs)

Inhibitory transmission was pharmacologically isolated by using a control bath solution containing ACSF with 6,7-dinitroquinoxaline-2,3-dione (DNQX) (40 µm) and D-2-amino-5-phosphonopentanoic acid (AP5) (50 µm) to block AMPA and NMDA glutamatergic transmission. Pipettes for pressure application of glycine were pulled to a resistance of ~1 MΩ (when filled with glycine solution [500 µm in ACSF containing DNQX and AP5]) and were visually guided near (~50 µm) the surface of a patched cell. Glycine was applied using ~2.5 psi pressure injection with a PLI 100A picoliter injector (Warner Instruments). Glycine application ranged from 10 ms to 10 s depending on the protocol. Pressure application of bath solution (ACSF) at 5 psi did not induce currents, suggesting mechanical artifacts did not contaminate recorded currents. For a few experiments, GABA was applied in the same manner (500 µm in ACSF containing DNQX and AP5).

IPSCs were evoked with 50 µs stimulus pulses with a stimulus isolation unit (Isoflex, A.M.P.I. Inc., Israel) through a concentric bipolar electrode with tungsten core (WPI TM53CCINS, Sarasota, FL). For recordings in the NM, the stimulation electrode was placed on fiber bundles adjacent to the nuclei in a ventrolateral location, and for the SON, a dorsomedial location was used. Presynaptic fibers were stimulated with pulse trains consisting of 15 pulses at 100 Hz. Stimulus magnitude (range 10–90 V) was gradually increased until IPSC amplitudes plateaued. The start of the 100 Hz train began when the current response to the 10 s glycine puff returned to baseline (usually within 5–8 s). After data were collected in the control condition, GlyRs were blocked by bath application of strychnine (1 µm) and data were collected again. Recovery of control values was attempted by washout of strychnine. This often took >20 min and full recovery was sometimes not attainable due to the high affinity binding of strychnine to the receptor. Peak IPSC amplitude during the train was used to compare treatment groups. In control, test (1 µm strychnine) and washout, evoked responses were compared between the no glycine condition and the glycine pre-pulse condition using the equation:

(1−(evoked amplitude with gly pre-pulse/evoked amplitude no gly)) × 100 = % suppression

**Figure 1:**
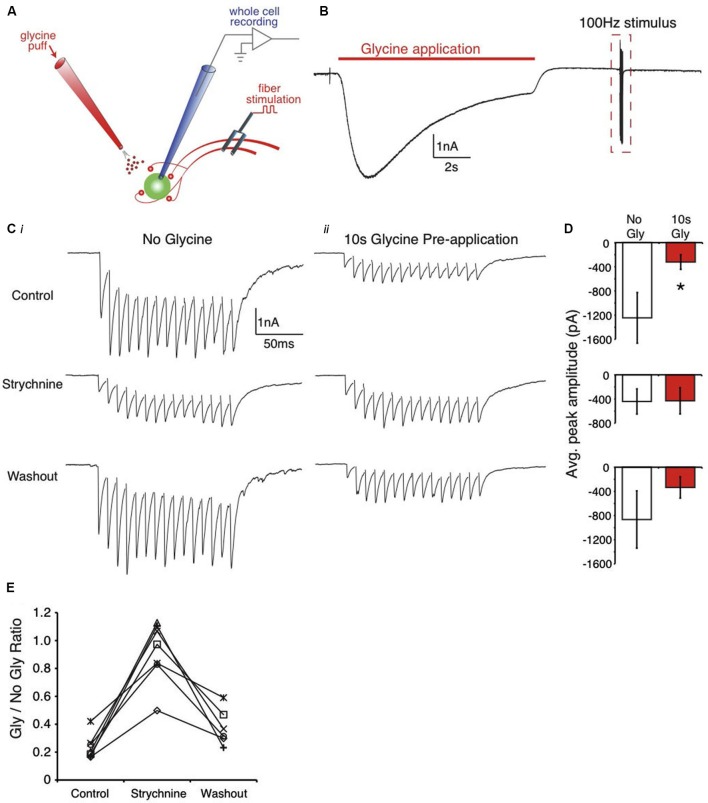
**Pre-application of glycine suppresses evoked IPSC trains in the SON**. **(A)** Schematic of the recording arrangement. **(B)** Representative trace from an E18 SON neuron during the 10 s glycine application. **(C)** Expanded view of the dashed box in **(B)** showing evoked responses. **(Ci)** Evoked responses in control, strychnine, and washout conditions with no glycine pre-application. **(Cii)** Evoked IPSCs following the glycine pre-application show amplitude suppression that is blocked by strychnine. **(D)** Population data values for the IPSC amplitudes in each condition (* indicates significant difference, *p* < 0.01). **(E)** Ratio of evoked amplitudes between glycine pre-application and no glycine in each condition reveals a decrease in the suppression (ratio near 1) in strychnine.

**Figure 2:**
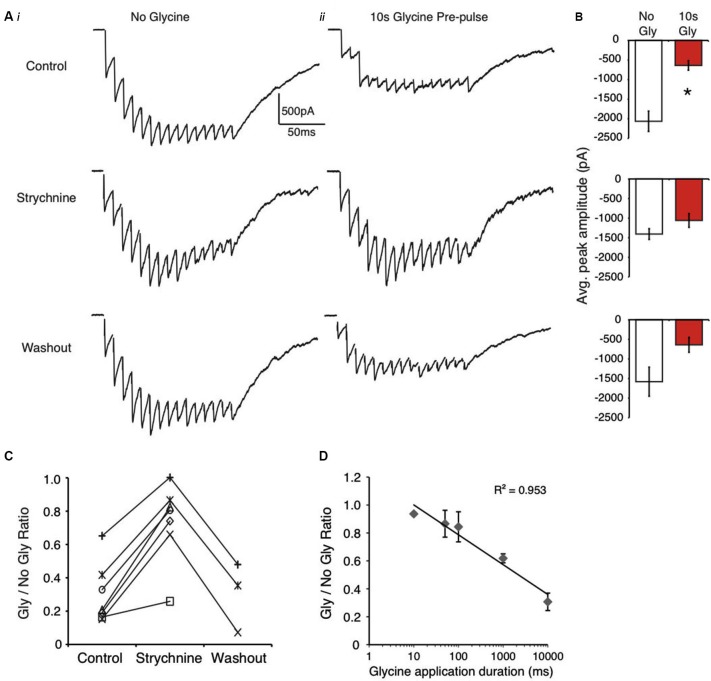
**Evoked IPSCs are suppressed by glycine pre-application in NM. (A)** Representative traces from an E18 neuron showing evoked responses without glycine **(Ai)** and with glycine pre-pulse **(Aii)** in control, strychnine and washout. **(B)** Population data of peak amplitude in the absence and presence of glycine pre-application in each condition (* indicates significant difference, *p* < 0.01). **(C)** Ratio of peak amplitude between Gly/no Gly shows an increased ratio value (less suppression) during GlyR block (strychnine). **(D)** Plot illustrates that IPSC suppression increases with increasing glycine application duration (*R*^2^ = 0.953, *p* < 0.001, Pearson’s Correlation).

This protocol and analysis was performed while holding the membrane voltage at three different potentials: −70 mV, approximating *V*_rest_ (Figures 1, 2); the reversal potential for glycine, ranging from −20 mV to −35 mV (average: −28.3 ± 5.2 mV, *n* = 6; derived empirically during the experiment) [Fig F3][Fig F4](Figure 5); and +10 mV, to drive the flux of Cl^−^ ions into the neuron (Figure 7). For these figures, data points are plotted as the quotient of the IPSC amplitude in the presence of glycine pre-application divided by IPSC amplitude in the absence of glycine (Gly/No Gly Ratio) in each condition.

**Figure 3 F3:**
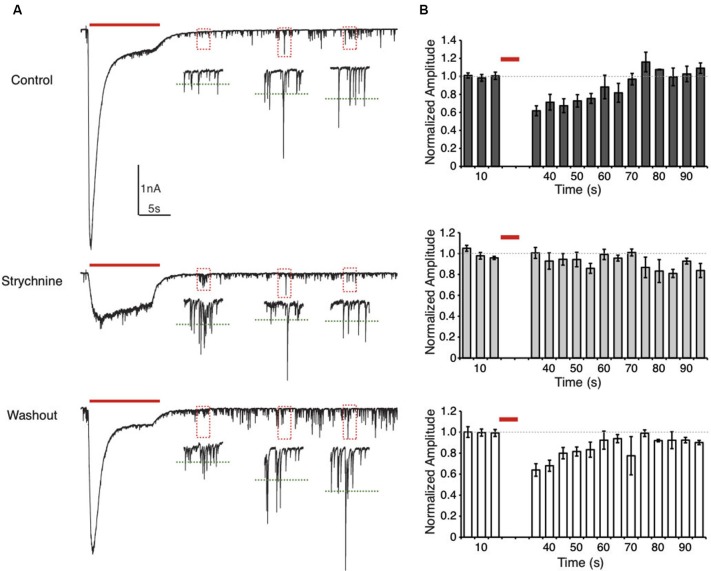
**Suppression of spontaneous IPSC (sIPSC) amplitudes after glycine application is eliminated by GlyR block. (A)** Representative traces from an E20 NM neuron showing sIPSCs recorded during a glycine application protocol. Insets are expanded views of the sIPSCs in each dashed box. The green dashed lines represent the average sIPSC amplitude of the events within this two-second window. In the control condition (top) sIPSC amplitude is suppressed for a short time after glycine application. This effect was not observed during GlyR block (strychnine) but recovered after washout. Note that in the strychnine condition the GlyR block was often incomplete, but the large onset current was eliminated. **(B)** Histograms representing the normalized population data for the sIPSC amplitude analysis. Dashed line represents the average sIPSC amplitude before the glycine application. Each bin represents the average sIPSC amplitude during a 5 s time window. In the control and washout condition, sIPSC amplitude recovered to baseline values after approximately 35 s.

**Figure 4 F4:**
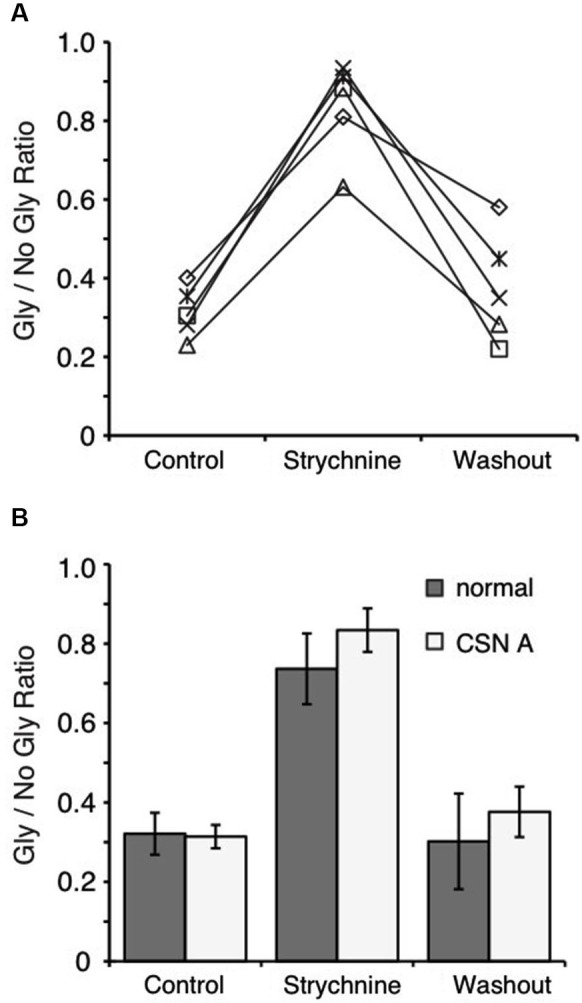
**Blocking phosphatase 2B activity does not affect suppression in the NM. (A)** Ratio of peak amplitude between Gly/no Gly in each condition reveals that inclusion of cyclosporin A in the recording pipette does not prevent suppression. **(B)** Population data comparing the results using internal solution with cyclosporin A (CSN A) and the normal internal (Figure 2C). Phosphatase 2B activity does not play a role in the observed suppression.

**Figure 5:**
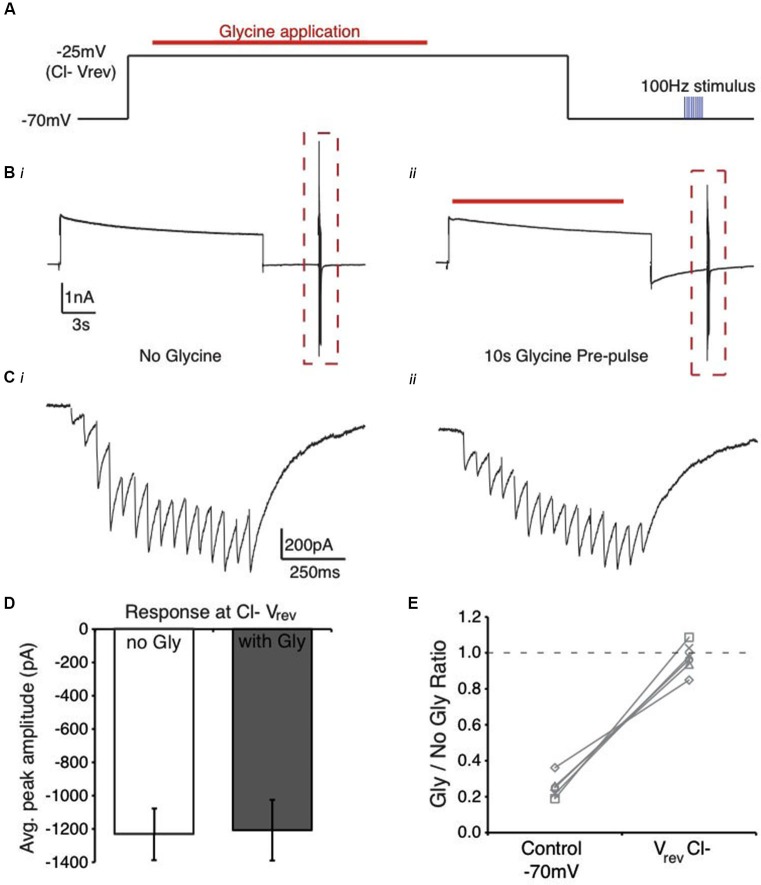
**Manipulation of Cl^−^ ion flux alters the suppression profile of glycine pre-application in the NM. (A)** Schematic of voltage clamp protocol where Cl^−^ flux is minimized by holding the membrane at the reversal potential for Cl^−^ during the glycine application. **(B)** Current response from protocol in **(A)** in the absence **(Bi)** and presence **(Bii)** glycine pre-application (red line). Note the similarity between the traces in **(Bi)** and **(Bii)**, suggesting minimal current due to glycine application. **(C)** Expanded view of evoked current responses from the boxed region in **(B)**. **(D)** Average peak amplitude of the evoked current for the population of cells tested at *V*_rev_ Cl^−^. **(E)** Ratio of peak amplitude between Gly/no Gly conditions in control and when glycine pre-application occurred at *V*_rev_ Cl^−^. Results were similar to glycine block (no suppression observed, Figure 2C). Dashed line represents a ratio of 1, indicating no suppression.

**Figure 6:**
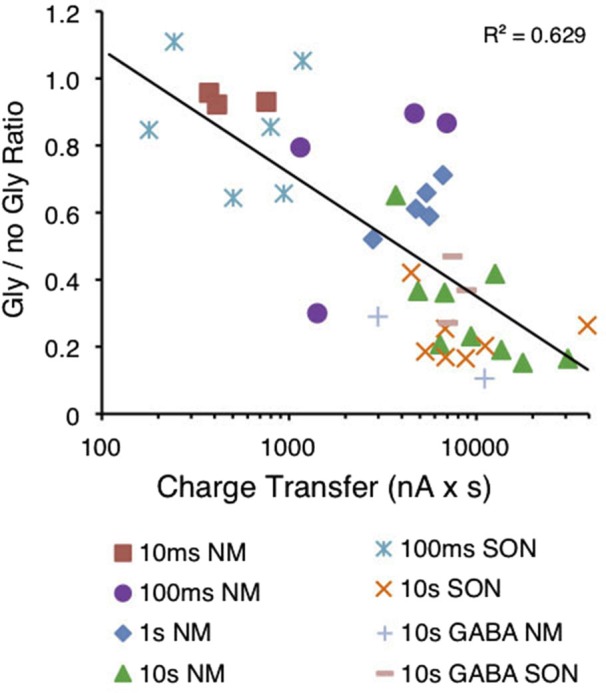
**Magnitude of suppression is correlated to the amount of charge transferred during puff application of glycine**. Graph depicting the normalized amplitude of the evoked IPSC (gly/no gly ratio) in relation to the amount of charge transferred in response to the glycine pre-application. A significant correlation was observed between the log of the charge transfer and suppression (*R*^2^ = 0.629, *p* < 0.0001, *n* = 40).

**Figure 7:**
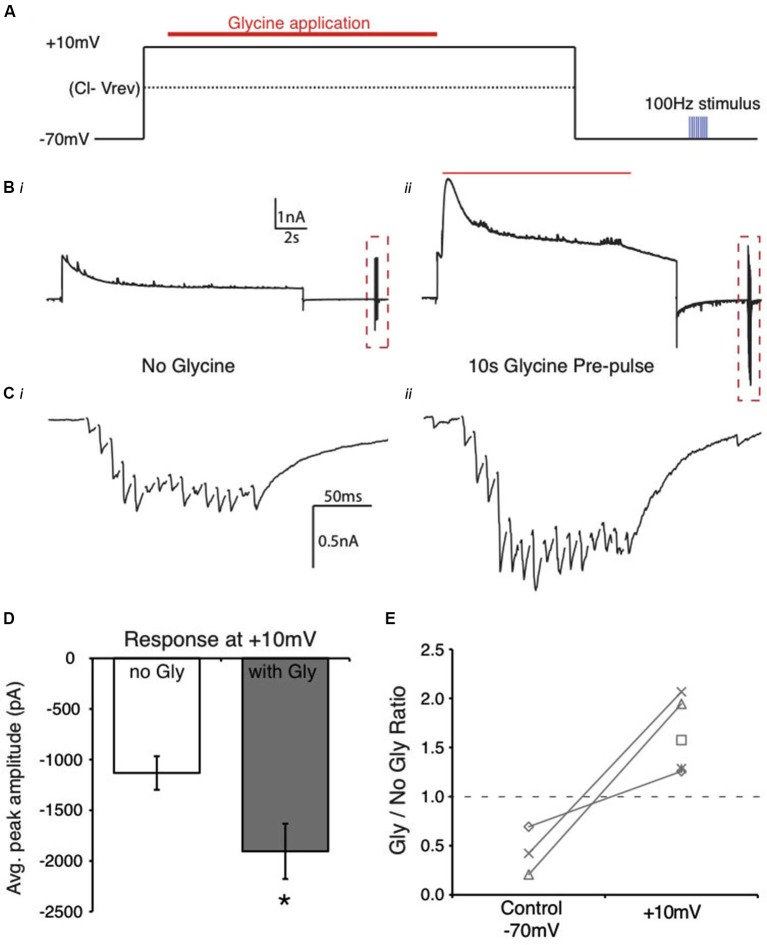
**Reversal of Cl^−^ ion flow results in an enhanced evoked IPSC in NM. (A)** Schematic of the flux reversal voltage clamp protocol. The membrane was held at approximately 45 mV positive to the *V*_rev_ Cl^−^. **(B)** Current response from an NM cell during the protocol in **(A)**. **(C)** Expanded view of evoked responses from **(B)** (dashed box, stimulus artifacts blanked for clarity). **Cii** shows the increase in evoked response after glycine application while holding at +10 mV. **(D)** Average peak amplitudes of the evoked IPSCs in the control (no gly) and in the presence of glycine pre-application (* indicates significant difference, *p* < 0.01). **(E)** Ratio of peak amplitude between Gly/no Gly conditions in control and when glycine pre-pulse occurred at +10 mV. The ratios were >1 in each cell when glycine application occurred during the +10 mV voltage step. Dashed line represents a ratio of 1, indicating no change in evoked amplitude.

The effect of GlyR activation on the amplitude of spontaneous IPSCs (sIPSCs) was also examined (Figure 3). A baseline amplitude of sIPSCs was acquired either during a 15 s interval prior to the application of glycine or during a 45 s recording preceding the protocol. After a 10 s glycine application, the current was allowed to return to baseline and then the amplitude of sIPSCs was measured. sIPSC amplitude was obtained for each event using a search template in Clampfit. sIPSC amplitudes were averaged during 5 s bins and compared to the pre-pulse average.

**Figure 8:**
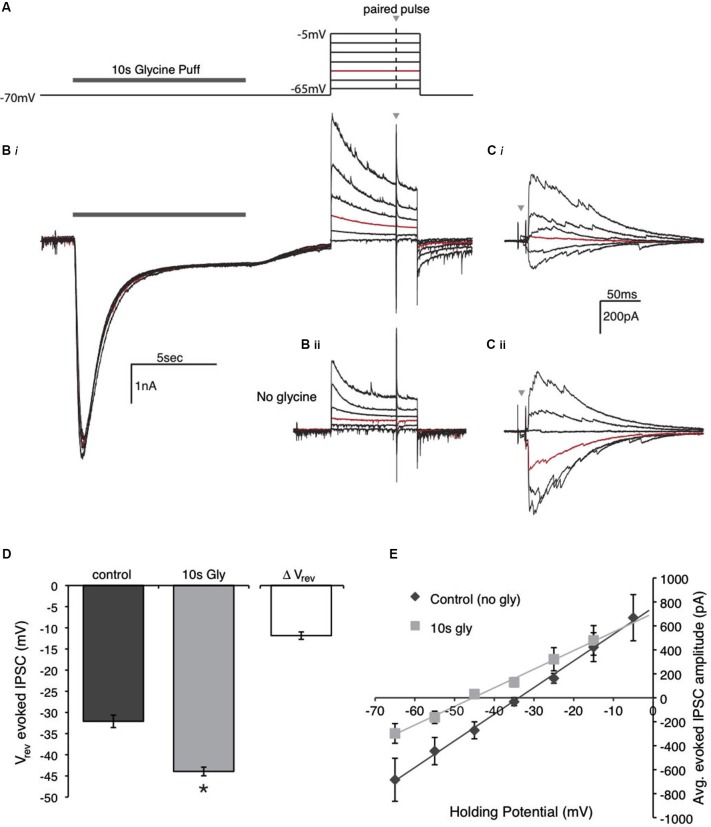
**Glycine application affects the driving force of evoked IPSCs by shifting the reversal potential. (A)** Schematic of the voltage clamp protocol used to compute reversal potential of evoked IPSCs. The protocol consisted of a paired pulse stimulus (gray arrow) to evoke IPSCs at various holding voltages in the presence or absence of the glycine pre-application. The highlighted (red) step at −45 mV is the voltage used for comparison in the Panels **(B)** and **(C)**. **(B)** Raw data traces recorded during the protocol in **(A)** in the presence **(Bi)** and absence **(Bii)** the glycine pre-application. **(C)** Expanded view of the IPSCs evoked with a paired pulse stimulus during each voltage step. Note that the red trace (*V*_hold_ = −45 mV) in **Ci** has an evoked IPSC with outward current, while the IPSC in **Cii** is inward. **(D)** Population data for the average reversal potential in the control and glycine pre-application conditions (* indicates significant difference, *p* < 0.001, *n* = 7) as well as the average change in reversal potential between the conditions. **(E)** IV plot constructed by averaging the absolute peak IPSC amplitude of the paired pulse at each voltage step for the population of neurons tested.

The magnitude of charge transfer (*I* × *t*) was measured during the glycine application using the area under the trace and analyzed using Clampfit. Reversal potential (*V*_rev_) of inhibition was computed by measuring the amplitude of evoked IPSCs at different voltages (range, −65 to −5 mV, protocol depicted in Figure 8A) and constructing an IV plot. *V*_rev_ was estimated by using a linear regression between the two voltages where the polarity of the IPSC changed from inward current to outward current. *V*_rev_ was calculated with and without a 10 s glycine pre-application.

Statistical significance was determined using one-way repeated measures ANOVA (Sigmaplot) unless otherwise stated. Data in the text is presented and mean ± SD. Error bars in figures are shown as SEM. Pearson’s correlation was used to determine significance for Figures 2D and 6.

## Results

### Glycine receptor (GlyR) transmission suppresses inhibitory postsynaptic current (IPSC) amplitude

The effect of GlyR activation on evoked IPSCs in the brainstem was evaluated by applying an exogenous puff of glycine preceding presynaptic fiber stimulation. The protocol consisted of a 10 s application of glycine (via a picoliter injector) followed by a 15 pulse, 100 Hz train of inhibitory presynaptic fiber stimulation with a bipolar tungsten electrode (Figure 1A depicts the recording arrangement). First, we performed this protocol on SON neurons where glycine represents about 1/2 of the IPSC amplitude and is co-released with GABA from inhibitory terminals (Coleman et al., 2011). Figure 1B shows an averaged response (3 traces) to glycine application. We compared the amplitude of the peak synaptically evoked IPSC with (Figure 1Cii), and without (Figure 1Ci), the glycine pre-application in control, and in the presence of the glycine antagonist, strychnine (example traces shown in Figure 1C). In the SON, a 10 s glycine pre-application resulted in approximately 75% suppression in the control condition (76.3 ± 8.9% suppression, mean ± SD, *n* = 7, *p* < 0.01, Figures 1C, D, *top*). The raw data averages for the evoked IPSC amplitudes are shown for each condition in Figure 1D. In every neuron tested, bath application of strychnine reduced the amount of suppression observed in the control condition (8.0 ± 22.2% suppression, *n* = 7, *p* > 0.05 vs. no glycine condition, Figures 1C, D, *middle*; *p* < 0.001 vs. control, Figure 1E). Suppression levels returned near control values after strychnine washout (62.2 ± 13.0% suppression, *n* = 6, *p* > 0.05, Figure 1E).

Next, we performed the same experiments in NM where glycinergic transmission is recruited during high frequency stimulation (Fischl et al., 2014). The results obtained using this protocol were similar to those observed in the SON (Figure 2) in most regards. The glycine pre-application significantly suppressed evoked IPSCs, a result that was reduced by blockade of GlyRs with strychnine in every neuron tested (control: 68.2 ± 15.8% suppression, *n* = 9, *p* < 0.001; strychnine: 26.3 ± 23.6% suppression, *n* = 7, *p* > 0.05 vs. no glycine condition, *p* < 0.001 vs. control; washout: 69.8 ± 20.9% suppression, *n* = 3, *p* > 0.05; Figures 2A, C). Average traces from a representative NM neuron are shown in Figure 2A. Population data averages for IPSC peak amplitudes are shown in Figure 2B. These results taken together with those obtained from SON neurons suggest that activation of GlyRs occludes inhibition mediated by GABA_A_ receptors.

In NM, also we investigated the time dependence of suppression by varying the duration of the agonist application from 10 ms to 10 s. Figure 2D shows that the degree of suppression was roughly linear with the log of the application duration, where longer application times lead to increased suppression (Figure 2D, *R*^2^ = 0.953, Pearson’s correlation, *p* < 0.001).

To further explore the temporal characteristics of this suppression, we measured the effect of prolonged glycine application on the amplitude of spontaneous IPSCs (sIPSCs) by comparing events pre- and post- glycine application (10 s pulse). This technique allowed us to evaluate the time course of recovery from suppression. In the control condition, sIPSC amplitude was suppressed by 38.2 ± 11.0% (*n* = 5) of pre-pulse levels when measured 10 s after the pulse. This was the earliest time point when the IPSCs could be accurately measured following the glycine response’s return to baseline (Figure 3). sIPSC amplitude recovered to 90% of pre-pulse amplitude after approximately 35 s (34.0 ± 11.4 s, *n* = 5). In contrast, in the presence of bath applied strychnine, there was no systematic change in sIPSC amplitude (*n* = 5). In several cells, the glycinergic response was not completely blocked with 1 µm strychnine (Figure 3A, *middle*). However, strychnine did eliminate the large amplitude onset current and minimized glycinergic currents resulting in unmodulated sIPSC amplitude for the population of cells tested (Figure 3B, *middle*). After washout, sIPSC amplitude suppression and recovery time course mirrored that of the control condition (36.0 ± 8.4% of pre-pulse levels, *n* = 4; recovery, 27.5 ± 10.4 s).

### Mechanism of suppression

In previous studies, several mechanisms have been shown to mediate GABA/glycine interactions at the postsynaptic neuron. Our results show that the suppression and recovery of IPSCs occurs over tens of seconds, suggesting that second messenger systems may influence receptor conductance. Li et al. (2003) found that phosphatase 2B activity was driven by GlyR activation and suppressed GABA_A_R currents in rat spinal cord neurons. We therefore tested whether the suppression that we observed was dependent on the phosphorylation state of the receptors by including cyclosporin A in the recording pipette to block phosphatase 2B activity. We observed the same suppression profile (Figure 4A) and no significant difference in suppression in the presence of cyclosporin A compared to the control internal solution (68.6 ± 6.6%, *n* = 5, *p* > 0.05, Figure 4).

Next, we determined if glycine binding to its receptor was sufficient to generate suppression, suggesting a biochemical process, or alternatively, if receptor binding and Cl^−^ flux was required. Neurons in NM maintain a high Cl^−^ concentration internally into maturity (~37 mM) yielding a Cl^−^ reversal potential around −34 mV (Monsivais and Rubel, 2001). For each neuron, we empirically derived the glycinergic reversal potential (average: −28.3 ± 5.2 mV, *n* = 6) using a brief (10 ms) glycine puff while manipulating the holding voltage to determine where the current was zero. To prevent Cl^−^ flux, we stepped the membrane voltage to the glycine conductance’s reversal potential during the glycine puff application (protocol shown in Figure 5A). This allowed receptor binding, but prevented transmembrane Cl^−^ movement (representative traces, Figures 5B, C). In this condition, the average IPSC response amplitude was nearly identical with or without glycine pre-pulse (Figure 5D, *n* = 6, *p* > 0.05) and suppression was eliminated (2.7 ± 8.1% suppression, *n* = 6, *p* < 0.001 vs. control [*V*_hold_ = −70 mV], Figure 5E). These results suggest that the glycine-induced suppression in this system likely depends on biophysical rather than biochemical factors. Specifically, they suggest that the glycine exerts its influence by disrupting the local Cl^−^ concentration gradient.

To evaluate whether the Cl^−^ gradient was disrupted by the GlyR activation, we measured the charge transfer during glycine puff application and compared it to the subsequent suppression. We predicted that if Cl^−^ flux were necessary for suppression, then greater charge transfer across the membrane would yield stronger suppression. Figure 6 shows glycine current area plotted against normalized evoked IPSC amplitude (Gly/No Gly ratio) for each cell tested in both NM and the SON. Similar to the results shown in Figure 2D, suppression was correlated to the log of the charge transfer (Figure 6, *R*^2^ = 0.629, *p* < 0.0001). An additional five cells are included for which GABA was used as the agonist. Puff application of GABA induced a similar amount of suppression (Figure 6, + and − symbols).

Given these results together with those observed in Figure 5, we hypothesized that manipulation of the driving force of Cl^−^ ions during the glycine application would directly influence the magnitude of suppression. We employed a voltage step protocol where Cl^−^ ion flux direction was inverted by holding the postsynaptic cell at a voltage positive to the predicted Cl^−^ reversal potential. We predicted that in this condition, since Cl^−^ flux would be inward, the glycine application would potentiate the evoked IPSCs. Indeed, when the membrane voltage was held at +10 mV during the glycine pulse, evoked IPSC amplitude increased significantly (62.6 ± 37.1% increase, *n* = 5, *p* < 0.05, Figures 7C–E). These results further implicate changes in the driving force of Cl^−^ ions as the most likely mechanism of glycinergic modulation of inhibitory current in this system.

Finally, these changes in driving force were confirmed by measuring the reversal potential of inhibitory conductances before and after agonist application (Figure 8). For this protocol, we first determined the reversal potential in the control condition by measuring the amplitude of evoked IPSCs at a range of holding voltages spanning the predicted Cl^−^ reversal potential (protocol depicted in Figure 8A). The response from a representative neuron is illustrated in Figure 8B. Figure 8Bi shows the response at each voltage when glycine was applied for 10 s preceding the voltage steps. An expansion of the absolute response magnitude is shown in Figure 8Ci where a *V*_rev_ around −45 mV (red trace) was observed for this neuron in this condition. Figures 8Bii and Cii show the response when no glycine was applied. Here, the *V*_rev_ was close to −34 mV (the predicted value with this internal solution). *V*_rev_ was interpolated for each cell, based on linear regression fits to the IV plot. For the population, the average *V*_rev_ for the evoked responses in the control condition was −32.1 ± 3.9 mV (Figures 8D, E; *n* = 7). When a 10 s glycine pulse preceded the voltage steps, a shift in the *V*_rev_ was observed in the negative direction (Figures 8Cii, D). The average *V*_rev_ in the test condition with glycine pre-application was −43.9 ± 3.3 mV (*n* = 7, *p* < 0.001). This shift was in the predicted direction following the outward flux of Cl^−^ in response to the glycine puff. These results indicate that GlyR activation and the resulting Cl^−^ flux alters the driving force of evoked IPSCs by shifting the reversal potential.

## Discussion

### Occlusion of inhibitory synaptic input by glycine receptor (GlyR) activation

Our data show that activation of GlyRs suppresses the amplitude of synaptically evoked IPSCs in NM and the SON. GABA and GlyRs are both permeable to Cl^−^ ions, and interactions between the two receptors have been documented in areas where both receptor types are present and activated via presynaptic transmitter release. Several studies have reported a similar occlusive effect that shows the amplitude of simultaneous application of GABA and glycine is less than the predicted summed amplitude of responses to each transmitter when applied individually (Trombley et al., 1999; Li et al., 2003). Further, in some cases, the occlusion is asymmetric between the transmitters. In two studies, pre-application of glycine occluded GABAergic currents to a greater degree than for the reverse (Li et al., 2003; Kalinina et al., 2009).

The proposed mechanisms that lead to such occlusion are diverse and include receptor level interactions (Barker and McBurney, 1979; Baev et al., 1992; Lewis and Faber, 1993; Trombley et al., 1999) and biochemical signaling cascades (Li et al., 2003). Alternatively, Karlsson et al. (2011) recently proposed that the occlusion is only an *apparent* cross-desensitization, and that the cross-suppression does not result from a change in channel conductance, but rather from local changes in the transmembrane Cl^−^ distribution (also concluded by Grassi, 1992). In our system, the changes in Cl^−^ ion concentration were sufficient to explain the observed occlusion. We saw no suppression when Cl^−^ flux was prevented and driving Cl^−^ flux into the cell resulted in increased evoked IPSC amplitudes, presumably due to increased driving force of Cl^−^. This conclusion was supported by the result that GABA and glycine pre-application were each similarly effective at generating suppression (Figure 6). However, this does not adequately explain asymmetric cross-inhibition seen in other studies where occlusion is attributed to the phosphorylation state of the receptors (Li et al., 2003). While we were unable to test the symmetry of the occlusion directly due to our study’s reliance on physiologically evoked IPSCs, our results ruled out phosphatase 2B activity as the mechanism of the observed suppression.

### Role of glycine in the avian brainstem

While a biochemical interaction between glycine and GABA receptors was not supported by our data, the hypothesis that glycinergic transmission in NM and the SON shapes overall inhibitory transmission remains a compelling possibility. Many studies in the avian sound localization circuit demonstrate modulatory mechanisms that dynamically alter inhibitory transmission. These mechanisms include activation of GABA_B_ receptors (Lu et al., 2005; Tang et al., 2009), metabotropic glutamate receptors (Lu, 2007; Okuda et al., 2013), and cooperation of both tonic and phasic inhibition (Tang et al., 2011; Yamada et al., 2013). GlyR activation could similarly modulate overall inhibitory strength.

GABA and glycine, either co-released or present at the same synapses, are also known to influence the kinetics of inhibition through a number of mechanisms. Postsynaptic activation of GlyRs and subsequent Cl^−^ movement would likely affect the Cl^−^ concentration gradient across the membrane. Changes in Cl^−^ concentration proximal to GABA and GlyR channel pores can modulate the temporal and voltage dependent properties of Cl^−^ currents (Moroni et al., 2011). Co-transmission of GABA with glycine has also been shown to speed up the decay kinetics of IPSCs in the mammalian cochlear nucleus (Lu et al., 2008). In the auditory brainstem, signal propagation is dependent on microsecond scale interaural differences in the arrival time of acoustic stimuli. The kinetics of both excitatory and inhibitory input to neurons that process these cues have an impact on their temporal selectivity (Kuba et al., 2005; Jercog et al., 2010; Fischl et al., 2012; Roberts et al., 2013). Therefore, small changes in kinetics caused by changes in local Cl^−^ gradients could modulate the integration of inputs that rely on precise timing in order to accurately localize sounds.

Glycinergic activity will also affect neurons differently depending on the physiology of the target cell. Physiological heterogeneity is a characteristic of neurons in both NA (Köppl and Carr, 2003; Kuo et al., 2009) and the SON (Coleman et al., 2011). In NA, the reversal potential for Cl^−^ is variable, such that some neurons were found to have a relatively depolarized *V*_rev_, and some, a hyperpolarized *V*_rev_ (Kuo et al., 2009). This suggests that the polarity of glycinergic transmission will also be dependent on neuron type. The Cl^−^ *V*_rev_ of SON neurons has not been thoroughly characterized, but one study using gramicidin perforated patch recordings observed an average Cl^−^ *V*_rev_ of −61 mV from data collected in three neurons (Monsivais and Rubel, 2001). Given the heterogeneity of response properties observed in the SON (Carr et al., 1989; Lachica et al., 1994; Coleman et al., 2011), a more thorough investigation of Cl^−^ regulation seems necessary to fully understand the role of inhibition in this circuit.

Our experiments add to the insights provided by several very recent studies that strive to understand the role of glycine in avian auditory processing. Glycine puff application may only approximate the physiological conditions that occur with intense, prolonged stimuli, where transmitters build up in the synapse and spillover into the extrasynaptic space. In our previous study, we found that glycine recruitment was highly dependent on input rate where the highest rate (200 Hz) resulted in the largest recruitment of glycinergic current in NM (Fischl et al., 2014). Whether this recruitment generally strengthens overall inhibition to maintain inhibitory tone (Fischl et al., 2014), or alternatively limits inhibition through occlusion as the current results suggest, requires further, *in vivo* experimentation.

## Summary

Numerous mechanisms have been identified that modulate inhibitory synaptic strength and influence computation in neural circuitry. These mechanisms are diverse in mode, site of action, and influence on signal propagation. One known mechanism of interest for synapses that co-release inhibitory transmitters, is the cross-modulatory suppression between GABA and GlyRs. In some cases, this suppression is clearly mediated by biochemical signaling pathways, while in other systems, the modulation appears to be related to biophysical mechanisms. We explored the nature of interactions between GABA- and glycinergic transmission in neurons that rely heavily on inhibition for precise computation, and for which glycinergic input has only recently been confirmed. We showed the influence of preceding receptor activation on evoked inhibitory transmission, where preceding GlyR activation consistently occluded evoked inhibitory transmission. The magnitude of the suppression was dependent on both the duration of agonist application and magnitude of charge transfer induced by glycine, or in a few cases, GABA. The glycine dependent occlusion was blocked in the presence of strychnine. Cl^−^ flux was necessary for occlusion suggesting that local changes in the Cl^−^ driving force resulted from the glycine treatment. Cross-suppressive interactions between GABA and GlyR channels at these synapses may provide an additional modulatory influence regulating inhibition in the avian sound localization circuit. Investigation of the role of glycine in NM and NL *in vivo* is necessary to determine whether these mechanisms impact transmission during sound evoked stimuli and if these modulations influence sound localization ability.

## Author contributions

Matthew J. Fischl and R. Michael Burger contributed to the conception and design of experiments as well as drafting and revising the manuscript. Matthew J. Fischl performed the experiments and analyzed the data. Matthew J. Fischl and R. Michael Burger approved the final version to be published.

## Conflict of interest statement

The authors declare that the research was conducted in the absence of any commercial or financial relationships that could be construed as a potential conflict of interest.

## References

[B1] Apostolides P. F. Trussell L. O. (2013). Rapid, activity-independent turnover of vesicular transmitter content at a mixed glycine/GABA synapse. J. Neurosci. 33, 4768–4781 10.1523/jneurosci.5555-12.2013 23486948 PMC3639006

[B2] Awatramani G. B. Turecek R. Trussell L. O. (2005). Staggered development of GABAergic and glycinergic transmission in the MNTB. J. Neurophysiol. 93, 819–828 10.1152/jn.00798.2004 15456797

[B3] Baev K. V. Rusin K. I. Safrono B. V. (1992). Primary receptor for inhibitory transmitters in lamprey spinal cord neurons. Neuroscience 46, 931–941 10.1016/0306-4522(92)90195-8 1311817

[B4] Barker J. L. McBurney R. N. (1979). GABA and glycine may share the same conductance channel on cultured mammalian neurones. Nature 277, 234–236 10.1038/277234a0 233114

[B5] Burger R. M. Fukui I. Ohmori H. Rubel E. W. (2011). Inhibition in the balance: binaurally coupled inhibitory feedback in sound localization circuitry. J. Neurophysiol. 106, 4–14 10.1152/jn.00205.2011 21525367 PMC3129726

[B6] Burger P. M. Hell J. Mehl E. Krasel C. Lottspeich F. Jahn R. (1991). GABA and glycine in synaptic vesicles: storage and transport characteristics. Neuron 7, 287–293 10.1016/0896-6273(91)90267-4 1678614

[B7] Carr C. E. Fujita I. Konishi M. (1989). Distribution of GABAergic neurons and terminals in the auditory system of the barn owl. J. Comp. Neurol. 286, 190–207 10.1002/cne.902860205 2794115

[B8] Chang E. H. Kotak V. C. Sanes D. H. (2003). Long-term depression of synaptic inhibition is expressed postsynaptically in the developing auditory system. J. Neurophysiol. 90, 1479–1488 10.1152/jn.00386.2003 12761279

[B9] Coleman W. L. Fischl M. J. Weimann S. R. Burger R. M. (2011). GABAergic and glycinergic inhibition modulate monaural auditory response properties in the avian superior olivary nucleus. J. Neurophysiol. 105, 2405–2420 10.1152/jn.01088.2010 21368002 PMC3094186

[B10] Eulenburg V. Armsen W. Betz H. Gomeza J. (2005). Glycine transporters: essential regulators of neurotransmission. Trends Biochem. Sci. 30, 325–333 10.1016/j.tibs.2005.04.004 15950877

[B11] Fischl M. J. Combs T. D. Klug A. Grothe B. Burger R. M. (2012). Modulation of synaptic input by GABAB receptors improves coincidence detection for computation of sound location. J. Physiol. 590, 3047–3066 10.1113/jphysiol.2011.226233 22473782 PMC3406390

[B12] Fischl M. J. Weimann S. R. Kearse M. G. Burger R. M. (2014). Slowly emerging glycinergic transmission enhances inhibition in the sound localization pathway of the avian auditory system. J. Neurophysiol. 111, 565–572 10.1152/jn.00640.2013 24198323 PMC3921398

[B13] Fukui I. Burger R. M. Ohmori H. Rubel E. W. (2010). GABAergic inhibition sharpens the frequency tuning and enhances phase locking in chicken nucleus magnocellularis neurons. J. Neurosci. 30, 12075–12083 10.1523/jneurosci.1484-10.2010 20826670 PMC3376706

[B14] Funabiki K. Koyano K. Ohmori H. (1998). The role of GABAergic inputs for coincidence detection in the neurones of nucleus laminaris of the chick. J. Physiol. 508, 851–869 10.1111/j.1469-7793.1998.851bp.x 9518738 PMC2230923

[B15] Gillespie D. C. Kim G. Kandler K. (2005). Inhibitory synapses in the developing auditory system are glutamatergic. Nat. Neurosci. 8, 332–338 10.1038/nn1397 15746915

[B16] Grassi F. (1992). Cl(-)-mediated interaction between GABA and glycine currents in cultured rat hippocampal neurons. Brain Res. 594, 115–123 10.1016/0006-8993(92)91035-d 1334762

[B17] Hassfurth B. Grothe B. Koch U. (2010). The mammalian interaural time difference detection circuit is differentially controlled by GABAB receptors during development. J. Neurosci. 30, 9715–9727 10.1523/jneurosci.1552-10.2010 20660254 PMC6632825

[B18] Jercog P. E. Svirskis G. Kotak V. C. Sanes D. H. Rinzel J. (2010). Asymmetric excitatory synaptic dynamics underlie interaural time difference processing in the auditory system. PLoS Biol. 8:e1000406 10.1371/journal.pbio.1000406 20613857 PMC2893945

[B20] Kalinina N. I. Kurchavyi G. G. Amakhin D. V. Veselkin N. P. (2009). Differences in the activation of inhibitory motoneuron receptors in the frog Rana ridibunda by GABA and glycine and their interaction. Neurosci. Behav. Physiol. 39, 775–783 10.1007/s11055-009-9192-9 19779830

[B21] Karlsson U. Druzin M. Johansson S. (2011). Cl(-) concentration changes and desensitization of GABA(A) and glycine receptors. J. Gen. Physiol. 138, 609–626 10.1085/jgp.201110674 22084415 PMC3226965

[B22] Köppl C. Carr C. E. (2003). Computational diversity in the cochlear nucleus angularis of the barn owl. J. Neurophysiol. 89, 2313–2329 10.1152/jn.00635.2002 12612008 PMC3259745

[B23] Kotak V. C. Sanes D. H. (2002). Postsynaptic kinase signaling underlies inhibitory synaptic plasticity in the lateral superior olive. J. Neurobiol. 53, 36–43 10.1002/neu.10107 12360581

[B24] Kotak V. C. Sanes D. H. (2003). Gain adjustment of inhibitory synapses in the auditory system. Biol. Cybern. 89, 363–370 10.1007/s00422-003-0441-7 14669016

[B25] Kuba H. Yamada R. Fukui I. Ohmori H. (2005). Tonotopic specialization of auditory coincidence detection in nucleus laminaris of the chick. J. Neurosci. 25, 1924–1934 10.1523/jneurosci.4428-04.2005 15728832 PMC6726073

[B26] Kuo S. P. Bradley L. A. Trussell L. O. (2009). Heterogeneous kinetics and pharmacology of synaptic inhibition in the chick auditory brainstem. J. Neurosci. 29, 9625–9634 10.1523/jneurosci.0103-09.2009 19641125 PMC2894706

[B27] Lachica E. A. Rubsamen R. Rubel E. W. (1994). GABAergic terminals in nucleus magnocellularis and laminaris originate from the superior olivary nucleus. J. Comp. Neurol. 348, 403–418 10.1002/cne.903480307 7844255

[B28] Lewis C. A. Faber D. S. (1993). GABA responses and their partial occlusion by glycine in cultured rat medullary neurons. Neuroscience 52, 83–96 10.1016/0306-4522(93)90184-h 8433811

[B29] Li Y. Wu L. J. Legendre P. Xu T. L. (2003). Asymmetric cross-inhibition between GABAA and glycine receptors in rat spinal dorsal horn neurons. J. Biol. Chem. 278, 38637–38645 10.1074/jbc.m303735200 12885784

[B30] Lu Y. (2007). Endogenous mGluR activity suppresses GABAergic transmission in avian cochlear nucleus magnocellularis neurons. J. Neurophysiol. 97, 1018–1029 10.1152/jn.00883.2006 17135473

[B31] Lu Y. Burger R. M. Rubel E. W. (2005). GABA(B) receptor activation modulates GABA(A) receptor-mediated inhibition in chicken nucleus magnocellularis neurons. J. Neurophysiol. 93, 1429–1438 10.1152/jn.00786.2004 15483063

[B49] Lu T. Rubio M. E. Trussell L. O. (2008). Glycinergic transmission shaped by the corelease of GABA in a mammalian auditory synapse. Neuron 57, 524–535 10.1016/j.neuron.2007.12.010 18304482

[B32] Lu T. Trussell L. O. (2000). Inhibitory transmission mediated by asynchronous transmitter release. Neuron 26, 683–694 10.1016/s0896-6273(00)81204-0 10896163

[B33] Magnusson A. K. Park T. J. Pecka M. Grothe B. Koch U. (2008). Retrograde GABA signaling adjusts sound localization by balancing excitation and inhibition in the brainstem. Neuron 59, 125–137 10.1016/j.neuron.2008.05.011 18614034

[B34] McIntire S. L. Reimer R. J. Schuske K. Edwards R. H. Jorgensen E. M. (1997). Identification and characterization of the vesicular GABA transporter. Nature 389, 870–876 10.1038/39908 9349821

[B35] Monsivais P. Rubel E. W. (2001). Accommodation enhances depolarizing inhibition in central neurons. J. Neurosci. 21, 7823–7830 11567073 10.1523/JNEUROSCI.21-19-07823.2001 PMC6762906

[B36] Monsivais P. Yang L. Rubel E. W. (2000). GABAergic inhibition in nucleus magnocellularis: implications for phase locking in the avian auditory brainstem. J. Neurosci. 20, 2954–2963 10751448 10.1523/JNEUROSCI.20-08-02954.2000 PMC6772202

[B37] Moroni M. Biro I. Giugliano M. Vijayan R. Biggin P. C. Beato M. (2011). Chloride ions in the pore of glycine and GABA channels shape the time course and voltage dependence of agonist currents. J. Neurosci. 31, 14095–14106 10.1523/jneurosci.1985-11.2011 21976494 PMC3204932

[B38] Okuda H. Yamada R. Kuba H. Ohmori H. (2013). Activation of metabotropic glutamate receptors improves the accuracy of coincidence detection by presynaptic mechanisms in the nucleus laminaris of the chick. J. Physiol. 591(Pt. 1), 365–378 10.1113/jphysiol.2012.244350 23090950 PMC3630791

[B39] Roberts M. T. Seeman S. C. Golding N. L. (2013). A mechanistic understanding of the role of feedforward inhibition in the mammalian sound localization circuitry. Neuron 78, 923–935 10.1016/j.neuron.2013.04.022 23764291 PMC3690812

[B40] Sagné C. El Mestikawy S. Isambert M. F. Hamon M. Henry J. P. Giros B. (1997). Cloning of a functional vesicular GABA and glycine transporter by screening of genome databases. FEBS Lett. 417, 177–183 10.1016/s0014-5793(97)01279-9 9395291

[B41] Takesian A. E. Kotak V. C. Sanes D. H. (2010). Presynaptic GABA(B) receptors regulate experience-dependent development of inhibitory short-term plasticity. J. Neurosci. 30, 2716–2727 10.1523/jneurosci.3903-09.2010 20164356 PMC3842473

[B42] Tang Z. Q. Dinh E. H. Shi W. Lu Y. (2011). Ambient GABA-activated tonic inhibition sharpens auditory coincidence detection via a depolarizing shunting mechanism. J. Neurosci. 31, 6121–6131 10.1523/jneurosci.4733-10.2011 21508237 PMC3090224

[B43] Tang Z. Q. Gao H. Lu Y. (2009). Control of a depolarizing GABAergic input in an auditory coincidence detection circuit. J. Neurophysiol. 102, 1672–1683 10.1152/jn.00419.2009 19571192 PMC2746784

[B44] Trattner B. Berner S. Grothe B. Kunz L. (2013). Depolarization-induced suppression of a glycinergic synapse in the superior olivary complex by endocannabinoids. J. Neurochem. 127, 78–90 10.1111/jnc.12369 23859596

[B45] Trombley P. Q. Hill B. J. Horning M. S. (1999). Interactions between GABA and glycine at inhibitory amino acid receptors on rat olfactory bulb neurons. J. Neurophysiol. 82, 3417–3422 10601472 10.1152/jn.1999.82.6.3417

[B46] Wojcik S. M. Katsurabayashi S. Guillemin I. Friauf E. Rosenmund C. Brose N. (2006). A shared vesicular carrier allows synaptic corelease of GABA and glycine. Neuron 50, 575–587 10.1016/j.neuron.2006.04.016 16701208

[B47] Yamada R. Okuda H. Kuba H. Nishino E. Ishii T. M. Ohmori H. (2013). The cooperation of sustained and phasic inhibitions increases the contrast of ITD-tuning in low-frequency neurons of the chick nucleus laminaris. J. Neurosci. 33, 3927–3938 10.1523/jneurosci.2377-12.2013 23447603 PMC6619327

[B48] Yang L. Monsivais P. Rubel E. W. (1999). The superior olivary nucleus and its influence on nucleus laminaris: a source of inhibitory feedback for coincidence detection in the avian auditory brainstem. J. Neurosci. 19, 2313–2325 10066281 10.1523/JNEUROSCI.19-06-02313.1999 PMC6782562

